# Attitudes toward gestational weight gain and exercise avoidance tendencies: a latent profile analysis and mediation test of internalized weight bias in pregnant women

**DOI:** 10.3389/fpubh.2026.1944847

**Published:** 2026-10-26

**Authors:** Wenmin Xie, Li Wang, Miaofang Ke, Meilan Ni

**Affiliations:** 1 Department of Obstetrics, The Maternal and Child Health Hospital, Xiamen, Fujian, China; 2 Department of Obstetrics and Gynecology, The Second Hospital of Zhangzhou, Zhangzhou, Fujian, China

**Keywords:** attitudes toward gestational weight gain, exercise avoidance tendency, internalized weight bias in pregnancy, personalized care, pregnant women

## Abstract

**Objective:**

This study aimed to examine the association between attitudes toward gestational weight gain and exercise avoidance tendencies among pregnant women from both variable-centered and person-centered perspectives. Specifically, it tested whether internalized weight bias in pregnancy statistically mediated this association and identified latent heterogeneous profiles of gestational weight-gain attitudes and their psychological and behavioral correlates.

**Methods:**

A cross-sectional survey design was adopted. A total of 433 pregnant women were recruited from March to May 2026. Measures included the Pregnancy and Weight Gain Attitude Scale, the Weight Bias Internalization Tool for Use in Pregnancy, the Tendency to Avoid Physical Activity and Sport Scale, and essential demographic information.

**Results:**

Attitudes toward gestational weight gain, internalized weight bias in pregnancy, and exercise avoidance tendencies were significantly and positively correlated with one another. The cross-sectional mediation model showed that attitudes toward gestational weight gain not only directly and positively was associated with exercise avoidance tendencies but also exerted a significant indirect association through internalized weight bias in pregnancy. Latent profile analysis supported the two-profile solution as the optimal model, classifying attitudes toward gestational weight gain into a “low concern profile” and a “high concern profile.” Pregnant women in the high concern profile reported significantly higher levels of internalized weight bias and exercise avoidance tendencies than those in the low concern profile.

**Conclusion:**

Negative attitudes toward gestational weight gain are closely associated with exercise avoidance tendencies among pregnant women, and this association was statistically mediated by internalized weight bias in pregnancy. Because these findings are cross-sectional, they highlight the need for future research to examine whether non-stigmatizing perinatal care that attends to attitudes toward gestational weight gain and offers individualized psychological support may help reduce exercise avoidance and promote maternal and infant wellbeing.

## 1 Introduction

Gestational weight gain is an important indicator reflecting physiological adaptation during pregnancy, fetal development, and both short- and long-term maternal and child health (1, 2). Previous studies have suggested that gestational weight gain is embedded within multiple psychosocial contexts, including pregnant women’s body image, maternal responsibility, perceived health risks, and social evaluation (3–5). For example, appropriate gestational weight gain contributes to placental function, fetal growth, and favorable pregnancy outcomes (6, 7). Conversely, both lower and higher gestational weight gain may be associated with a range of maternal and infant health outcomes, including preterm birth, fetal growth restriction, and gestational diabetes mellitus (8, 9). Some studies have also reported relationships between gestational weight gain and children’s subsequent body size (10, 11). However, existing research has predominantly examined weight-related behaviors during pregnancy from the perspectives of nutritional intake, physical activity, and clinical considerations (12, 13), while relatively little attention has been paid to pregnant women’s attitudes toward gestational weight gain itself. Even fewer studies have examined the relationship between attitudes toward gestational weight gain and exercise avoidance.

In fact, attitudes toward gestational weight gain do not simply reflect a unidimensional positive or negative evaluation. Rather, they may simultaneously involve rational acknowledgment of the necessity of weight gain, emotional distress regarding bodily changes, a sense of responsibility for fetal health, and anticipated pressure related to postpartum recovery (14, 15). These attitudinal components may form distinct psychological response patterns and further influence whether pregnant women are willing to adopt health-promoting behaviors during pregnancy, particularly whether they engage in regular and appropriate physical activity. Therefore, this study examines the relationship between attitudes toward gestational weight gain and exercise avoidance from both person-centered and variable-centered perspectives. Such an approach may not only enhance understanding of individual differences in weight-related attitudes and health behaviors during pregnancy but also provide a theoretical basis for precise perinatal psychological support programs and health education.

Internalized weight bias in pregnancy refers to the extent to which pregnant women accept negative weight stereotypes as self-relevant and apply them to themselves—that is, self-devaluation, shame, and the belief that one is less worthy or attractive because of weight gained during pregnancy. It is conceptually distinct from experienced or enacted weight stigma, which denotes encountering negative stereotypes, derogatory comments, or unfair treatment from others (e.g., health professionals, family, or peers) because of one’s weight (3, 16). Due to rapid bodily changes, weight gain, and normative societal expectations regarding the “ideal” pregnant body, pregnant women may be particularly vulnerable to weight-related evaluation and body surveillance (4). However, gestational weight gain is simultaneously framed by medical discourse as an indicator for health monitoring and by sociocultural discourse as a matter of appearance management and moralized self-discipline. Specifically, weight gain above recommendations may be morally interpreted as a failure of personal control (17), whereas insufficient weight gain may be judged as irresponsibility toward the fetus. This dual pressure requires pregnant women to accept bodily growth in order to support fetal development while also potentially fearing appearance-related devaluation and social criticism associated with weight gain.

More importantly, weight stigma (i.e., experienced or enacted stigma) may originate not only from healthcare professionals, family members, and peers but also from media-promoted weight-loss narratives, pregnancy body-management content, and idealized images of rapid postpartum recovery (18, 19). Once stigma is internalized, pregnant women may transform external negative evaluations into self-criticism, leading to shame, anxiety, and body dissatisfaction, thereby altering their cognitive appraisal of weight gain and subsequent behavioral responses (20, 21). Therefore, this study further examines the association between internalized weight bias in pregnancy and attitudes toward gestational weight gain.

Exercise is an essential component of health management during pregnancy (22) and has been widely shown to improve cardiopulmonary function, support healthy gestational weight gain, reduce the risk of pregnancy complications, and facilitate postpartum recovery (23). Nevertheless, in real-world settings, many pregnant women do not meet recommended levels of physical activity (24), and exercise avoidance tendency represents an important psychological barrier to exercise participation during pregnancy. Exercise avoidance may manifest as overestimation of exercise-related risks, concerns about physical discomfort, fear regarding fetal safety, sensitivity to others’ evaluations, and avoidance of body exposure in exercise settings (25, 26).

For pregnant women who experience weight stigma or hold negative attitudes toward weight gain, exercise may be perceived as a tool for weight control and health maintenance; however, exercise environments may also intensify body comparison, appearance anxiety, and perceived evaluation by others (27). This negatively charged meaning of exercise may weaken exercise motivation, increase anticipatory anxiety before exercise, and promote avoidance of exercise-related contexts (28). In addition, pregnancy-related physical discomfort, fatigue, and safety concerns may themselves reduce willingness to exercise (29). When combined with self-monitoring and emotional burden caused by weight stigma, pregnant women may be more likely to engage in sedentary behaviors, reduce outdoor activities, or avoid public exercise settings (30). Accordingly, this study investigates the role of internalized weight bias in pregnancy in the relationship between attitudes toward gestational weight gain and exercise avoidance tendencies.

Although previous studies have separately explored associations among gestational weight gain, body image, weight stigma, and physical activity, most have relied on variable-centered approaches to examine linear relationships between average levels of these variables and health behaviors (15, 31). While such approaches are useful for revealing overall trends, they may obscure substantial heterogeneity within the population of pregnant women. Latent profile analysis can be used to identify heterogeneity in attitudes toward gestational weight gain and further examine differences in internalized weight bias and exercise avoidance tendencies across different attitudinal profiles. This may help clarify which pregnant women are more likely to develop negative, ambivalent, or defensive exercise avoidance tendencies. Such findings can identify priority populations for perinatal health promotion programs and services and clarify the psychological correlates, including internalized weight bias—that co-occur with exercise avoidance tendencies.

### 1.1 Theoretical framework and conceptual model

To integrate these constructs, we draw on three complementary theoretical perspectives. First, the COBWEBS model (32) proposes that weight stigma is a psychologically stressful experience that, once internalized, promotes maladaptive coping and behavioral avoidance, which in turn reinforce further weight concern in a self-perpetuating cycle. Second, internalized weight bias theory (33, 34) distinguishes internalized weight bias—the self-application of negative weight stereotypes—from experienced or enacted stigma and from overt discriminatory treatment, and specifies that internalized bias, rather than external discrimination alone, drives shame, diminished self-efficacy, and avoidance of health-promoting behaviors such as exercise. Third, the principle of reciprocal determinism from Social Cognitive Theory (35) frames attitudes (cognitive factors), internalized self-evaluations (personal factors), and exercise behavior as mutually reinforcing. Applied to pregnancy, these perspectives jointly predict that negative attitudes toward gestational weight gain predispose women to internalize weight-related negative self-evaluations, which in turn manifest as exercise avoidance (Figure 1).

**Figure 1:**
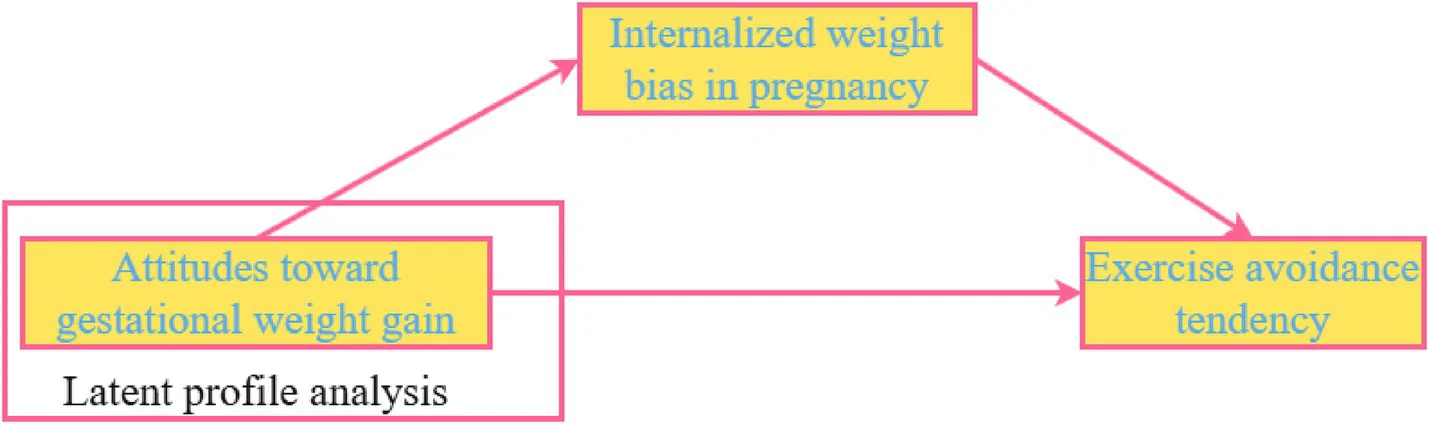
Conceptual framework diagram.

Based on this framework, we derived the following hypotheses:

*H1*: more negative attitudes toward gestational weight gain are positively associated with exercise avoidance tendency.

*H2*: more negative attitudes are positively associated with internalized weight bias.

*H3*: internalized weight bias is positively associated with exercise avoidance tendency and statistically mediates the attitude–avoidance association.

*H4*: multiple latent profiles of gestational weight-gain attitudes exist, and women in higher-concern profiles report greater internalized weight bias and exercise avoidance tendency.

### 1.2 Why weight concern may inhibit rather than promote exercise

A potential objection is that weight concern should logically motivate weight-control behavior, including exercise, rather than avoidance. Three mechanisms resolve this apparent paradox. First, anticipatory anxiety and threat appraisal: when exercise settings heighten body visibility and social evaluation, weight concern is experienced as an evaluative threat rather than an approach motive, so the anticipatory affect it elicits is avoidance-oriented (28). Second, body surveillance and objectification: women who hold negative weight attitudes are more likely to anticipate being watched and judged, transforming exercise into a stage for body exposure rather than a self-care behavior, which increases avoidance (36). Third, behavioral inhibition of internalized weight bias: the COBWEBS model specifies that internalized bias elicits shame and reduced self-efficacy—inhibitory states that suppress, rather than activate, health-promoting behavior (32, 34). Weight concern therefore promotes weight-control behavior only when experienced with high self-efficacy and low social-evaluative threat; when accompanied by shame and anticipated judgment, avoidance predominates.

### 1.3 Rationale for the dual variable- and person-centered analytic strategy

The variable-centered mediation analysis addresses how the attitude–avoidance association operates on average—specifically, whether internalized weight bias statistically mediates it—whereas the person-centered latent profile analysis addresses who is at elevated risk, by testing whether subpopulations with qualitatively different attitude patterns exist. The two methods cross-validate one another: if person-centered profiles that differ in attitude level also differ in internalized weight bias and avoidance in the expected direction, this corroborates the variable-centered pathway using an independent analytic logic. Unlike a median split, latent profile analysis treats profile membership as a probabilistic latent classification estimated from the full 16-item response pattern: it avoids a single artificial cut-point, incorporates measurement uncertainty via posterior probabilities, can detect shape differences (non-parallel profiles) that level-based grouping cannot, and is validated against formal fit statistics (LMR, BLRT) and substantive interpretability.

### 1.4 Internalized weight bias and exercise behavior during pregnancy: cross-sectional and longitudinal evidence

Emerging evidence clarifies the relationship between weight-related self-evaluation and exercise. Qualitative research on larger-bodied pregnant women shows that internalized weight bias is a salient barrier to prenatal physical activity (31). Prospective studies demonstrate that body image satisfaction mediates the association between exercise behavior and depressive symptoms and gestational weight gain (37). Importantly, recent longitudinal studies using the random-intercept cross-lagged panel model (RI-CLPM) show bidirectional, dynamic associations between weight self-stigma and physical exercise at both between-person and within-person levels (38, 39). These findings imply that the relationship is plausibly bidirectional rather than unidirectional. Because the present study is cross-sectional, we can estimate associational—not temporal—paths; we situate our findings within this longitudinal evidence in the Discussion.

## 2 Methods

### 2.1 Study design

This study adopted a cross-sectional survey design to examine the relationships among attitudes toward gestational weight gain, internalized weight bias in pregnancy, and exercise avoidance tendencies, and to further identify latent heterogeneous profiles of attitudes toward gestational weight gain.

This study was reviewed and approved by the Human Research Ethics Committee of Xiamen Maternity and Child Health Hospital (No. KY-2026-057-K01). All research procedures complied with ethical principles for medical research. Data were collected using anonymous coding, and the principle of data confidentiality was emphasized. All participants were required to read and sign the informed consent form before completing the questionnaire.

### 2.2 Participants

From March to May 2026, participants were recruited from two tertiary hospitals in Fujian Province using convenience sampling. Specifically, the researchers contacted the heads of the obstetrics and gynecology departments of the collaborating hospitals and provided detailed explanations of the study purpose, potential risks, and benefits. After approval was obtained, the researchers introduced the study purpose, content, participation procedures, and estimated time required to eligible pregnant women in obstetric outpatient waiting areas or at maternal health education sessions.

For women willing to participate, the researchers provided a QR code for the electronic questionnaire or instructions for completing the paper-based questionnaire. Participants completed the questionnaire independently after fully reading the informed consent information. After questionnaire completion, the researchers checked for missing values and asked participants with incomplete responses to complete the missing items on site.

To further enhance the accuracy of the study, strict inclusion and exclusion criteria were established. The inclusion criteria were as follows: (1) age ≥18 years; (2) clinically confirmed pregnancy, including the first, second, or third trimester; (3) ability to understand the Chinese questionnaire; (4) voluntary participation in the study and provision of informed consent.

The exclusion criteria were as follows: (1) severe pregnancy complications for which complete avoidance of exercise had been advised by a physician, such as severe cardiopulmonary disease, active bleeding, severe threatened miscarriage, or severe placental abnormalities; (2) previous or current diagnosis of a severe mental disorder, cognitive impairment, or neurological disease; (3) severe physical disease or high-risk pregnancy conditions that could substantially affect weight change, psychological experience, or exercise behavior; (4) patterned responses or an excessively short response time of less than 3 min.

During data collection, the researchers approached 468 potential participants. Thirty-five participants were excluded: 6 were younger than 18 years, 6 had pregnancy complications, and 23 showed patterned responses. Therefore, the final valid sample included 433 participants, with an effective response rate of 92.52%.

### 2.3 Measures tools

#### 2.3.1 Demographic information

Demographic information was collected, including age, gestational week, height, pre-pregnancy weight, current weight, pre-pregnancy body mass index, current body mass index, educational level, monthly income, and other relevant characteristics.

“Pregnancy complication” was a self-reported, binary physician-diagnosed item (gestational diabetes mellitus, gestational hypertension, placenta previa, threatened miscarriage, or other conditions recorded in the antenatal record). “Adverse pregnancy history” was any self-reported prior miscarriage, preterm birth, or stillbirth. Participants with severe complications contraindicating exercise were excluded, so the complication covariate reflects non-contraindicating conditions and perceived health risk rather than absolute exercise restriction.

#### 2.3.2 Pregnancy and weight gain attitude scale

Attitudes toward gestational weight gain were assessed using items from the Pregnancy and Weight Gain Attitude Scale developed by Dipietro, Millet (40). The original scale consists of 16 items covering two domains: attitudes toward pregnancy weight gain (13 items) and behaviors related to pregnancy weight gain (3 items). A sample item is: “Do you agree that women should be especially careful not to get fat during pregnancy?” In the present study, this scale was used to assess attitudes toward gestational weight gain among Chinese pregnant women. Responses were rated on a 5-point Likert scale ranging from 1 = “I do not feel this way at all” to 5 = “I feel this way very strongly.” Negatively worded attitude items were reverse-scored so that higher scores uniformly indicate greater weight-gain concern/negative attitude; the 3 behavioral items were scored in the same direction (higher = more weight-control behavior). The total score ranged from 16 to 80, with higher scores indicating stronger weight gain-related concerns, worries, or negative attitudes. In this study, Cronbach’s alpha for the scale was 0.917, indicating good internal consistency.

Dimensionality: a unidimensional CFA (AMOS 29.0) fit the data (χ^2^/df = 2.953, CFI = 0.951, RMSEA = 0.067, TLI = 0.937). Because the scale is theoretically two-dimensional (13 attitude + 3 behavior items), we also fit a two-factor model; it fit significantly better than the one-factor model (Δχ^2^ = 310.19, Δdf = 1, *p* < 0.001), with a high interfactor correlation (r = 0.516). As a sensitivity analysis, the mediation was re-run using a 13-item attitude-only score. We retained the 16-item unidimensional total for the primary analysis because the behavioral items did not materially affect the results and the unidimensional total is the standard scoring for this scale (Supplementary Table S1).

#### 2.3.3 Internalized weight bias in pregnancy scale

Internalized weight bias in pregnancy was measured using items from the Weight Bias Internalization Tool for Use in Pregnancy developed by Nagpal, Pearce (41). The scale contains 10 items and is unidimensional. A sample item is: “Do you agree that you are not as attractive as other pregnant people because of the weight you have gained during pregnancy?” In the present study, this scale was used to assess internalized weight bias among Chinese pregnant women. The WBI-TP is a purpose-built, pregnancy-adapted measure; its selection reflects the documented psychometric limitations of generic self-report weight-stigma measures. Responses were rated on a 5-point Likert scale ranging from 1 = “strongly disagree” to 5 = “strongly agree.” Item 8 was reverse-scored before being included in the total score. The total score ranged from 10 to 50, with higher scores indicating a higher level of internalized weight bias during pregnancy. In this study, Cronbach’s alpha for the scale was 0.919, indicating good internal consistency. A unidimensional model of internalized weight bias in pregnancy was constructed using AMOS 29.0, and the model demonstrated good fit, as shown in Table 1. Formal cross-cultural validation of the 5-point format is recommended in future work.

**Table 1:** Confirmatory factor analysis of core study variables.

Variables	χ^2^/df	GFI	AGFI	RMSEA	CFI	TLI
Pregnancy and weight gain attitude	2.953	0.925	0.892	0.067	0.951	0.937
Internalized weight bias in pregnancy	2.795	0.958	0.933	0.064	0.975	0.967
Exercise avoidance tendency	1.264	0.985	0.972	0.025	0.997	0.996

#### 2.3.4 Exercise avoidance tendency scale

Exercise avoidance tendency was measured using the Tendency to Avoid Physical Activity and Sport Scale developed by Fan, Chang (42). The scale consists of 9 items and is unidimensional. A sample item is: “Do you agree that you find yourself avoiding physical activity and sport because of your weight concerns?” In this study, the scale was used to assess exercise avoidance tendencies among Chinese pregnant women. Responses were rated on a 5-point Likert scale ranging from 1 = “strongly disagree” to 5 = “strongly agree.” No items were reverse-scored. The total score ranged from 9 to 45. Higher scores indicated a stronger tendency to avoid physical activity and sport due to weight- or appearance-related concerns. In this study, Cronbach’s alpha for the scale was 0.894, indicating good internal consistency. A unidimensional model of exercise avoidance tendency was constructed using AMOS 29.0, and the model demonstrated good fit, as shown in Table 1.

### 2.4 Statistical analysis

Statistical analyses were performed using SPSS 27.0, Mplus 8.3, and AMOS 29.0. The significance level was set at α = 0.05. First, routine analyses were conducted, including reliability testing, confirmatory factor analysis, common method bias testing, descriptive statistics, correlation analysis, and between-group comparisons. Next, PROCESS Model 4 was used to construct a mediation model with internalized weight bias in pregnancy as the mediator (43). The mediating effect was tested using the bootstrap method with 5,000 resamples, and 95% confidence intervals were calculated for the indirect effect. If the confidence interval did not include 0, the mediating effect was considered statistically significant.

Subsequently, standardized item mean scores of attitudes toward gestational weight gain were used as profile indicators. The number of latent profiles was increased sequentially starting from a one-profile model, and the model fit and classification quality of different solutions were compared. Model selection was based on the Akaike information criterion, Bayesian information criterion, sample-size-adjusted Bayesian information criterion, entropy, Lo–Mendell–Rubin likelihood ratio test, bootstrap likelihood ratio test, and the sample proportion of each latent class (44–46). In addition, the determination of latent profiles considered theoretical interpretability, reasonable class size, and clinical relevance, in order to avoid selecting profiles composed of very small samples that were difficult to interpret. Finally, after the optimal latent profile model was determined, participants were assigned to corresponding profiles based on their maximum posterior probabilities. Differences in internalized weight bias in pregnancy and exercise avoidance tendencies across the identified gestational weight gain attitude profiles were then compared.

The mediation was re-estimated with HC3 robust standard errors and bias-corrected bootstrap (5,000 resamples); a 13-item attitude-only sensitivity analysis was also run. A post-hoc sensitivity analysis was conducted for the mediation model, and a Monte Carlo simulation assessed the adequacy of *N* = 433 for a two-profile LPA.

## 3 Results

### 3.1 Demographic characteristics

A total of 433 pregnant women were included in the final analysis. Detailed demographic information is presented in Table 2. The mean age was 32.410 ± 3.533 years and mean gestational age 25.410 ± 6.714 weeks. Mean pre-pregnancy BMI was 18.677 ± 2.552 kg/m^2^ and mean current BMI 24.770 ± 3.109 kg/m^2^. Two hundred and twenty-five (52.0%) participants had a BMI below 18.5, 202 (46.7%) had a BMI between 18.5 and 24.9, 4 (0.9%) had a BMI between 25.0 and 29.9, and 2 (0.5%) had a BMI at or above 30. The sample was thus concentrated at the lower end of the BMI distribution. Mean gestational weight gain was 15.5 ± 7.4 kg at a mean 25.4 weeks; this is high but plausible given that most participants began pregnancy with a BMI below 18.5, and weight was self-reported. Height was self-reported in whole centimeters (17 distinct values, 152–171 cm), yielding a small SD (2.449 cm).

**Table 2:** Demographic information of participants.

Variables	Items	Number (N)	Proportion (%)
Educational background	Primary school and below	59	13.6%
	Middle school - High school	100	23.1%
	Bachelor degree or above	274	63.3%
Monthly income level	¥4,000 or less	73	16.9%
	4,001–8,000¥	191	44.1%
	8,001¥ or more	169	39.0%
Employment status	Full time job	180	41.6%
	Part time	108	24.9%
	Unemployment	55	12.7%
	Take maternity leave	90	20.8%
Parity	Primipara	279	64.4%
	Multipara	154	35.6%
Adverse pregnancy history	Yes.	255	58.9%
	No.	178	41.1%
Pregnancy complication	Yes.	268	61.9%
	No.	165	38.1%
Age (years)	M ± SD	32.410 ± 3.533	
Gestational weeks	M ± SD	25.410 ± 6.714	
Pre-pregnancy weight (kg)	M ± SD	47.614 ± 6.883	
Height (cm)	M ± SD	159.650 ± 2.449	
Current weight (kg)	M ± SD	63.115 ± 8.031	
Pre-pregnancy BMI	M ± SD	18.677 ± 2.552	
Current BMI	M ± SD	24.770 ± 3.109	

### 3.2 Common method bias

Because the primary study variables were assessed using self-report questionnaires, there was a potential risk of common method bias. Therefore, Harman’s single-factor test was conducted as a preliminary assessment of common method bias. The results showed that an unrotated exploratory factor analysis extracted four factors with eigenvalues greater than 1, and the first factor accounted for 35.646% of the total variance, which was below the critical threshold of 40%. These findings indicate that no single factor explained the majority of the common variance, suggesting that serious common method bias was unlikely to be present in the study data.

A single-factor CFA in which all 35 items loaded on one factor fit poorly (χ^2^ = 5009.37, df = 560, CFI = 0.33, RMSEA = 0.136, SRMR = 0.149), whereas the three-factor model fit substantially better (χ^2^ = 2668.97, df = 557, CFI = 0.58, RMSEA = 0.094, SRMR = 0.131), with a significant difference (Δχ^2^ = 2340.40, Δdf = 3, *p* < 0.001). No single common-method factor explained the data.

### 3.3 Descriptive statistics and correlation analysis

The descriptive statistics and correlation analyses for the core study variables are presented in Tables 3, 4. The skewness values of the core variables ranged from −1.136 to −0.958, and the kurtosis values ranged from −0.458 to −0.025. According to the criteria proposed by Kline (47), these values were within an acceptable range, indicating that the data approximately conformed to a normal distribution.

**Table 3:** Descriptive statistics of the core study variables.

Variables	M	SD	Skewness	Kurtosis	Range	IQR
Pregnancy and weight gain attitude	3.636	0.777	−0.958	−0.458	1.69–4.56	1.19
Internalized weight bias in pregnancy	3.631	0.891	−1.136	−0.236	1.20–4.70	1.20
Exercise avoidance tendency	3.614	0.904	−1.035	−0.025	1.00–4.89	1.33

**Table 4:** Correlations between the core study variables and continuous variables.

Variables	1	2	3	4	5	6	7	8	9	10
1. Pregnancy and weight gain attitude	1									
2. Internalized weight bias in pregnancy	0.471**	1								
3. Exercise avoidance tendency	0.500**	0.688**	1							
4. Age	0.035	−0.012	0.006	1						
5. Gestational weeks	−0.088	−0.142**	−0.187**	0.022	1					
6. Pre-pregnancy weight	−0.113*	−0.189**	−0.227**	0.085	0.041	1				
7. Height	0.026	0.001	−0.03	−0.037	0.009	0.220**	1			
8. Current weight	−0.028	0.004	−0.038	0.078	0.003	0.510**	0.098*	1		
9. Pre-pregnancy BMI	−0.121*	−0.194**	−0.223**	0.096*	0.038	0.973**	−0.006	0.491**	1	
10. Current BMI	−0.032	0.006	−0.027	0.085	0.001	0.439**	−0.147**	0.969**	0.475**	1

*p < 0.05, **p < 0.01.

The correlation analysis showed that attitudes toward gestational weight gain were significantly and positively correlated with internalized weight bias in pregnancy (r = 0.471, *p* < 0.01). Internalized weight bias in pregnancy was significantly and positively correlated with exercise avoidance tendency (r = 0.688, *p* < 0.01). In addition, attitudes toward gestational weight gain were also significantly and positively correlated with exercise avoidance tendency (r = 0.500, *p* < 0.01).

Discriminant validity: AVE was 0.563 (attitudes), 0.755 (internalized weight bias), and 0.772 (avoidance); every squared latent-factor correlation (max = 0.343) was below the smallest AVE (Fornell–Larcker criterion satisfied). The HTMT ratio between internalized weight bias and avoidance was 0.766 (< 0.85), supporting discriminant validity. VIF values for the two focal predictors were 1.29 (attitudes) and 1.37 (internalized weight bias), indicating no harmful multicollinearity.

### 3.4 Between-group analysis of exercise avoidance tendency

To further identify differences in exercise avoidance tendency across demographic and obstetric characteristics, exercise avoidance tendency was used as the dependent variable, and between-group comparisons were conducted according to educational level, monthly income, employment status, parity, adverse pregnancy history, and pregnancy complications (Table 5).

**Table 5:** Differences in exercise avoidance tendency by demographic and obstetric characteristics.

Variables	Items	M	SD	F/t	*p*	η^2^/Cohen d
Educational background	Primary school and below	3.763	0.837	0.921	0.399	0.004
	Middle school - High school	3.596	0.832			
	Bachelor degree or above	3.589	0.943			
Monthly income level	¥4,000 or less	3.696	0.803	5.579	0.004	0.025
	4,001–8,000¥	3.741	0.814			
	8,001¥ or more	3.436	1.011			
Employment status	Full time job	3.530	1.009	1.098	0.350	0.008
	Part time	3.682	0.783			
	Unemployment	3.743	0.893			
	Take maternity leave	3.622	0.820			
Parity	Primipara	3.612	0.903	−0.068	0.945	−0.007
	Multipara	3.618	0.909			
Adverse pregnancy history	Yes.	3.635	0.874	0.565	0.572	0.055
	No.	3.585	0.947			
Pregnancy complication	Yes.	3.724	0.816	3.274	0.001	0.324
	No.	3.435	1.009			

The results showed that exercise avoidance tendency did not differ significantly by educational level (*F* = 0.921, *p* = 0.399), employment status (*F* = 1.098, *p* = 0.350), parity (t = −0.068, *p* = 0.945), or adverse pregnancy history (t = 0.565, *p* = 0.572). However, significant differences in exercise avoidance tendency were observed across monthly income levels (*F* = 5.579, *p* = 0.004) and pregnancy complication status (t = 3.274, *p* = 0.001).

### 3.5 Mediation test of internalized weight bias

PROCESS Model 4 was used to examine the mediating role of internalized weight bias in pregnancy in the association between attitudes toward gestational weight gain and exercise avoidance tendency, as shown in Table 6. Gestational week, pre-pregnancy BMI, pre-pregnancy weight, monthly income, and pregnancy complications were included as covariates.

**Table 6:** Regression models for internalized weight bias in pregnancy.

Model	Model 1	Model 2	
Predictive variable	Attitudes toward gestational weight gain	Attitudes toward gestational weight gain	Internalized weight bias in pregnancy
Outcome variable	Internalized weight bias in pregnancy	Exercise avoidance tendency	Exercise avoidance tendency
b	0.500	0.254	0.557
SE	0.048	0.044	0.039
t	10.355	5.800	14.164
*p*	<0.001	<0.001	<0.001
95% CI (b)	[0.405, 0.595]	[0.168, 0.341]	[0.480, 0.635]
β	0.436	0.219	0.549
R	0.517	0.730	
R^2^	0.268	0.533	
F	25.953***	69.175***	

***p < 0.001; b = unstandardized regression coefficient; β = standardized regression coefficient; t = test statistic for the significance of each path coefficient; R = multiple correlation coefficient; R^2^ = proportion of variance explained in the dependent variable; F = overall model fit statistic from the regression ANOVA. The 95% CI reported is the bias-corrected bootstrap confidence interval for the indirect (mediation) effect, based on 5,000 bootstrap resamples. Mediation is considered significant if this interval does not contain zero.

The results showed that, in Model 1, attitudes toward gestational weight gain significantly and positively was associated with internalized weight bias in pregnancy (b = 0.500, SE = 0.048, t = 10.355, *p* < 0.001; β = 0.436). The explanatory power of Model 1 was R^2^ = 0.268, and the overall model was significant (*F* = 25.953).

In Model 2, attitudes toward gestational weight gain was a significant positive associated with exercise avoidance tendency (b = 0.254, SE = 0.044, t = 5.800, *p* < 0.001; β = 0.219). Meanwhile, internalized weight bias in pregnancy also significantly and positively was associated with exercise avoidance tendency (b = 0.557, SE = 0.039, t = 14.164, *p* < 0.001; β = 0.549). The explanatory power of Model 2 was R^2^ = 0.533, and the overall model was significant (*F* = 69.175).

The bootstrap mediation analysis results are presented in Table 7. The total association of attitudes toward gestational weight gain on exercise avoidance behavior was 0.533 (SE = 0.048, 95%CI = [0.440, 0.627]). The direct association of attitudes toward gestational weight gain on exercise avoidance behavior was 0.254 (SE = 0.044, 95%CI = [0.168, 0.341]), accounting for 47.65% of the total effect.

**Table 7:** Decomposition of the mediating effect of internalized weight bias in pregnancy.

Type of effect	Effect	SE	LLCI	ULCI	Proportion of effect
Total effect	0.533	0.048	0.440	0.627	100.00%
Direct effect	0.254	0.044	0.168	0.341	47.65%
Indirect effect	0.279	0.039	0.207	0.358	52.35%

Further analysis indicated that the cross-sectional indirect effect through the pathway of attitudes toward gestational weight gain → internalized weight bias in pregnancy → exercise avoidance behavior was 0.279 (SE = 0.039, 95%CI = [0.207, 0.358]), accounting for 52.35% of the total association.

Because both direct and indirect associations were significant and in the same direction, this indicates partial, not full, mediation. Robust (HC3) standard errors were only slightly larger (path a: 0.055; path b: 0.045; direct: 0.047) and did not alter significance. A sensitivity analysis excluding the 3 behavioral items (13-item attitude score) yielded a nearly identical indirect association (0.247, 95% CI [0.180, 0.323]; 52.4%) (Supplementary Table S2). The near-identical proportions with (52.4%) and without (54.9%) covariates suggest limited influence of the selected covariates, underscoring the need to measure additional confounders in future work.

### 3.6 Robustness test without covariates

To examine the stability of the mediation model, the mediation analysis was repeated without including covariates, as shown in Table 8. The results showed that the total association in the model without covariates was 0.581 (SE = 0.049, 95%CI = [0.486, 0.677]). The direct association of attitudes toward gestational weight gain on exercise avoidance behavior was 0.263 (SE = 0.044, 95%CI = [0.175, 0.350]).

**Table 8:** Decomposition of the mediating effect of internalized weight bias in pregnancy without covariates.

Type of effect	Effect	SE	LLCI	ULCI	Proportion of effect
Total effect	0.581	0.049	0.486	0.677	100.00%
Direct effect	0.263	0.044	0.175	0.350	45.27%
Indirect effect	0.319	0.043	0.238	0.405	54.91%

Further analysis showed that the cross-sectional indirect effect through the pathway of attitudes toward gestational weight gain → internalized weight bias in pregnancy → exercise avoidance behavior was 0.319 (SE = 0.043, 95%CI = [0.238, 0.405]). Because the 95% confidence interval for the indirect association did not include 0, the mediating role of internalized weight bias in pregnancy remained significant in the model without covariates.

In terms of effect proportion, the indirect association accounted for 54.91% of the total association in the model without covariates, whereas the direct association accounted for 45.27%. These findings were generally consistent with the results obtained after controlling for gestational week, pre-pregnancy BMI, pre-pregnancy weight, monthly income, and pregnancy complications, indicating that the mediating effect of internalized weight bias in pregnancy in the association between attitudes toward gestational weight gain and exercise avoidance tendency was relatively robust.

### 3.7 Latent profile analysis of attitudes toward gestational weight gain

To identify latent heterogeneity in attitudes toward gestational weight gain, standardized item mean scores of the Pregnancy and Weight Gain Attitude Scale were used as profile indicators. Latent profile models with one to five classes were fitted sequentially, and the model fit indices are presented in Table 9. The results showed that as the number of classes increased from one to five, the AIC, BIC, and adjusted BIC generally decreased, suggesting that adding classes improved model fit (Figure 2).

**Table 9:** Model fit indices for the one- to five-class latent profile models.

Profile	AIC	BIC	aBIC	Entropy	LMR (p)	BLRT (p)	Proportion
Profile 1	21807.948	21938.211	21836.661	—	—	—	—
Profile 2	**18451.183**	**18650.649**	**18495.150**	**0.998**	**<0.001**	**<0.001**	**26.79%/73.21%**
Profile 3	17853.170	18121.839	17912.392	0.997	0.981	<0.001	26.33%/63.97%/9.70%
Profile 4	17293.321	17631.192	17367.796	0.995	0.001	<0.001	17.32%/9.24%/9.47%/63.97%
Profile 5	17049.148	17456.222	17138.877	0.997	0.009	<0.001	13.86%/9.01%/3.69%/9.47%/63.97%

Bold represents the fit index of the optimal latent profile.

**Figure 2:**
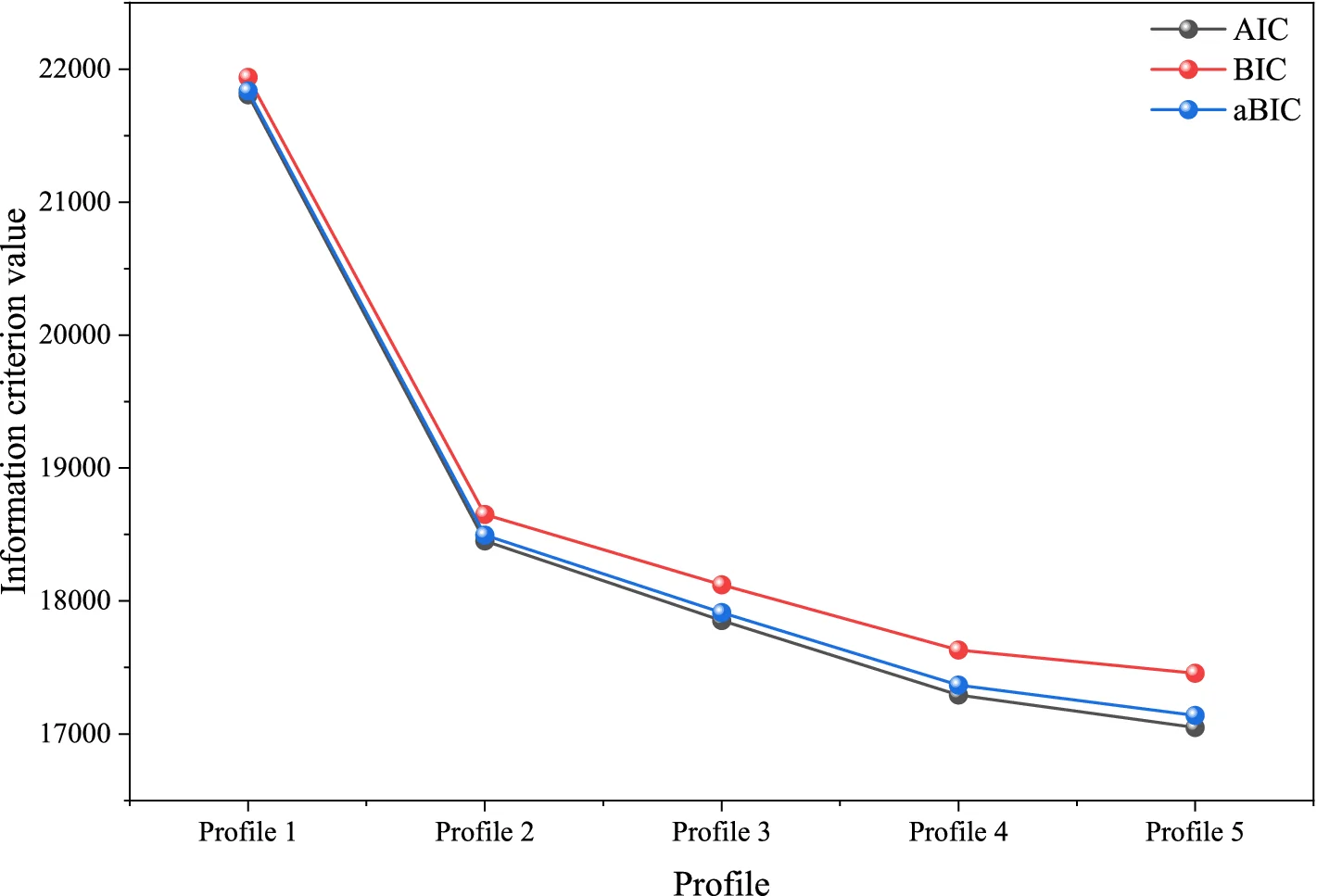
Elbow plot of AIC/BIC/aBIC across 1–5 profile solutions.

For the three-class model, the AIC, BIC, and adjusted BIC decreased further, and entropy was 0.997. However, the Lo–Mendell–Rubin likelihood ratio test was not significant (*p* = 0.981), suggesting that the three-class model did not significantly improve model fit compared with the two-class model.

For the four-class model, the AIC, BIC, and adjusted BIC continued to decrease, entropy was 0.995, and both the LMR and BLRT were significant. However, this solution included two relatively small classes, accounting for 9.24 and 9.47% of the sample, respectively. Although the five-class model showed lower AIC, BIC, and adjusted BIC values, with entropy of 0.997 and significant LMR and BLRT results, one class included only 3.69% of the sample. This suggested potential over-extraction of classes or instability of a very small class. Therefore, the two-class model was ultimately selected as the optimal latent profile model. This model not only demonstrated extremely high classification accuracy entropy = 0.998, but also yielded two classes with adequate proportions, accounting for 26.79 and 73.21% of the sample, respectively.

The average posterior classification probability matrix showed that individuals assigned to Class 1 had an average probability of 0.999 of being classified into Class 1, whereas individuals assigned to Class 2 had an average probability of 1.000 of being classified into Class 2. These findings indicate that the two-class model had very clear classification boundaries and high individual classification reliability (Table 10).

**Table 10:** Average posterior classification probabilities for the two-class latent profile model.

Class	Class 1	Class 2
Class 1	0.999	0.001
Class 2	0.000	1.000

Although the four-class solution’s smallest classes (9.24 and 9.47%) are technically acceptable (>5%, *n* > 25), and the five-class solution’s smallest class (3.69%, *n* ≈ 16) falls below the reliability threshold, the two-profile solution was retained because it yields two large, interpretable, clinically actionable groups with no loss of substantive information and because the three-class solution did not significantly improve fit (LMR *p* = 0.981). The three-profile solution (a third, non-parallel “high-concern/low-acceptance” profile, 9.70%) is reported as a sensitivity analysis (Supplementary Table S4).

Based on the above results, a profile plot of the two-class solution was generated according to the mean patterns of the indicators of attitudes toward gestational weight gain, as shown in Figure 3. Item means with SE and 95% CI for the two-profile solution (Supplementary Table S3).

Class 1 was labeled the “low concern profile.” This class accounted for approximately 26.79% of the sample and was characterized by lower risk levels related to attitudes toward gestational weight gain, suggesting less shame, anxiety, or resistance regarding pregnancy-related weight gain.

Class 2 was labeled the “high concern profile.” This class accounted for approximately 73.21% of the sample and was characterized by higher levels of weight-related concern, distress about bodily changes, and negative evaluation tendencies regarding gestational weight gain (Figure 3).

**Figure 3:**
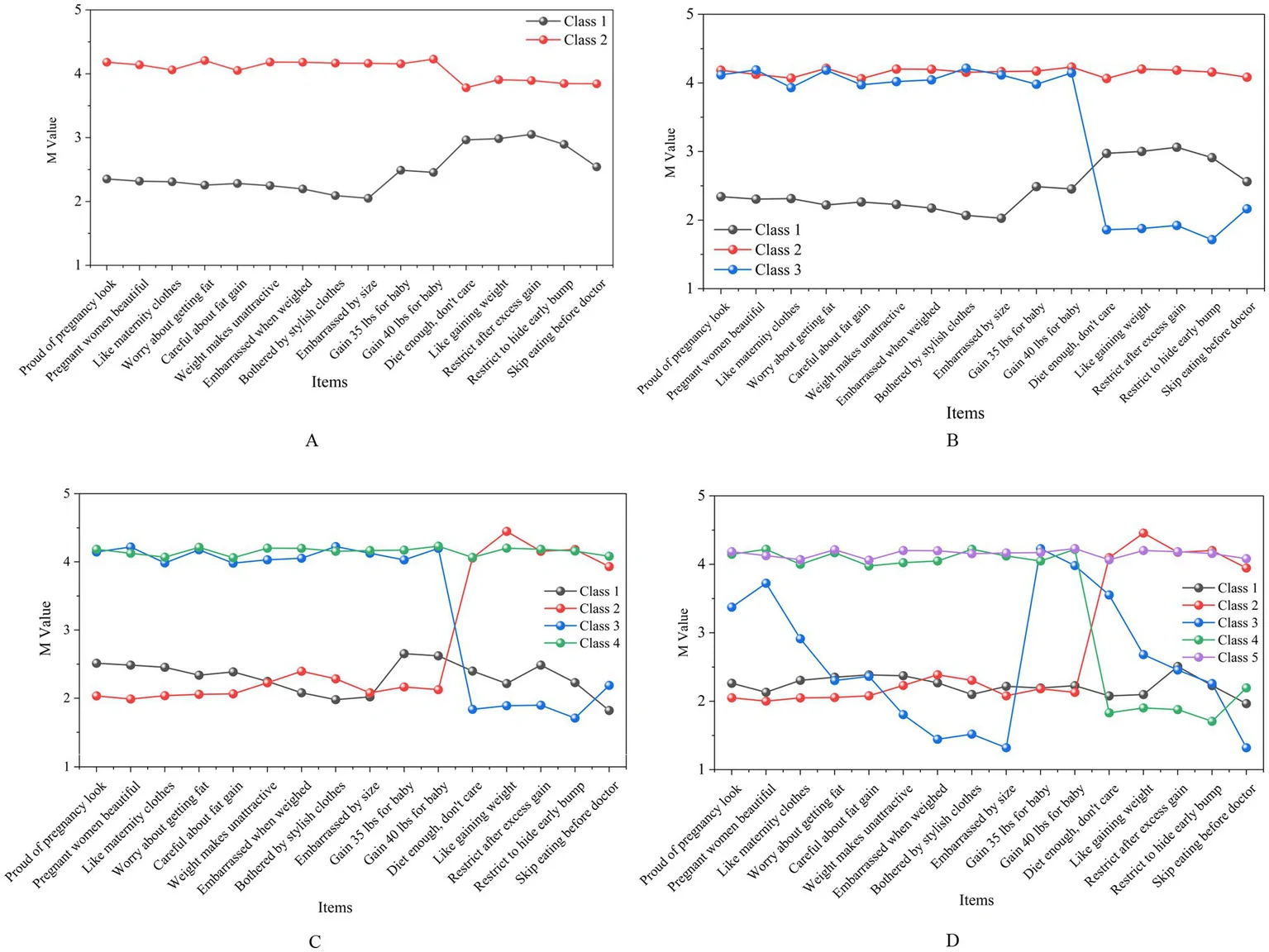
Four latent profile models. **(A)** Profile plot of the two-class latent profile solution; **(B)** Profile plot of the three-class latent profile solution; **(C)** Profile plot of the four-class latent profile solution; **(D)** Profile plot of the five-class latent profile solution.

### 3.8 Differences across the optimal latent profiles

After identifying the two-class model as the optimal latent profile solution, differences in internalized weight bias in pregnancy and exercise avoidance tendency were further compared between the two attitude profiles, as shown in Table 11.

**Table 11:** Differences in internalized weight bias and exercise avoidance tendency across the optimal latent profiles.

Class	Internalized weight bias in pregnancy		Exercise avoidance tendency	
	M	SD	M	SD
Class 1	2.97	1.038	2.894	1.06
Class 2	3.874	0.688	3.878	0.669
t	−10.456		−11.441	
*p*	<0.001		<0.001	
Cohen d	−1.135		−1.241	

The results showed significant differences between Class 1 and Class 2 in both internalized weight bias in pregnancy (t = −10.456, *p* < 0.001) and exercise avoidance tendency (t = −11.441, *p* < 0.001). Pregnant women in the high concern profile reported significantly higher levels of internalized weight bias and exercise avoidance tendency than those in the low concern profile. These findings suggest that heterogeneity in attitudes toward gestational weight gain can effectively identify pregnant women at elevated risk of exercise avoidance.

## 4 Discussion

### 4.1 Variable-centered analysis

This study found a significant positive association between attitudes toward gestational weight gain and exercise avoidance tendency. Pregnant women’s attitudes toward weight gain are not merely subjective evaluations of bodily changes; they may also further influence health behavior choices (48, 49). Previous studies have suggested that gestational weight gain is an important component of fetal development, placental function maintenance, and maternal physiological adaptation (6, 50). However, weight gain may also bring about appearance changes, physical discomfort, pressure regarding postpartum recovery, and concerns about being evaluated by others.

When pregnant women hold higher levels of negative concern, anxiety, or ambivalent attitudes toward weight gain, exercise situations may be endowed with a stronger sense of threat. For example, pregnant women may worry about body shape exposure, awkward movements, or negative evaluation from others during exercise, and may interpret exercise as a compulsory behavior for weight control (51). Therefore, attitudes toward gestational weight gain may increase exercise avoidance tendency by intensifying pre-exercise anxiety, reducing exercise self-efficacy, and reinforcing body surveillance.

This study also found that internalized weight bias in pregnancy had a significant cross-sectional mediating effect in the association between attitudes toward gestational weight gain and exercise avoidance tendency. Negative attitudes toward gestational weight gain may predispose pregnant women to internalize weight-related negative evaluations (i.e., internalized weight bias), making them more likely to experience shame, body dissatisfaction, and a sense of being judged. These negative psychological experiences may further reduce their willingness to engage in exercise and increase avoidance of exercise-related situations.

According to internalized weight bias theory (33, 34) and the COBWEBS model (32), internalized weight bias may co-occur with greater psychological distress, lower self-esteem, and reduced self-efficacy, all of which have been linked to physical-activity avoidance (52, 53). For pregnant women, the psychological burden associated with internalized weight bias may be more complex, because exercise involves not only personal physical performance but also fetal safety, maternal responsibility, and others’ evaluations (54). Therefore, when pregnant women report stronger internalized weight bias, even if they are aware of the health benefits of moderate exercise, they may reduce exercise due to shame, safety concerns, or appearance anxiety.

According to weight stigma theory (55), weight stigma may lead individuals to avoid physical activity by increasing psychological stress, lowering self-esteem, and weakening self-efficacy. For pregnant women, the psychological burden caused by weight stigma may be more complex, because exercise involves not only personal physical performance but also fetal safety, maternal responsibility, and others’ evaluations. Therefore, when pregnant women perceive stronger weight stigma, even if they are aware of the health benefits of moderate exercise, they may reduce exercise due to shame, safety concerns, or appearance anxiety.

Pre-pregnancy BMI showed small but significant negative associations with attitudes toward gestational weight gain (r = −0.121), internalized weight bias (r = −0.194), and exercise avoidance tendency (r = −0.223), a pattern opposite to the positive BMI–weight-concern associations typically reported in pregnancy research (56, 57). We interpret this as a consequence of restriction of range: 52.0% of the sample had a BMI below 18.5 before pregnancy and only ~1.4% had a BMI of 25 or above, so the upper end of the BMI distribution—where weight bias and avoidance are most strongly expressed in general populations—is almost entirely absent. Within this truncated, low-BMI sample, the small negative associations should not be interpreted as evidence that lower BMI causes greater weight-related concern. Rather, the findings indicate that women who begin pregnancy at the lower end of the BMI distribution can still experience substantial weight-related concern. At the same time, the restricted BMI range in the sample may have reduced the study’s ability to detect stronger positive associations that have been observed in samples with a broader distribution of BMI values. Because the present study included relatively few participants at the upper end of the BMI distribution, the extent to which these findings apply across the full range of pregnant populations remains uncertain.

Quantitative comparison with prior evidence. The 52.4% indirect-association proportion is higher than the 20–45% typically reported in weight-stigma mediation studies in other populations, a difference we attribute to the conceptual proximity of internalized weight bias to avoidance during pregnancy, ceiling-constrained distributions, and cross-sectional self-report estimation, which tends to overestimate indirect associations relative to longitudinal designs. Our exercise-avoidance mean (3.614 ± 0.904) is comparable to or modestly higher than TAPAS means reported in Chinese college-student samples (42), consistent with heightened weight/appearance concern during pregnancy. The two profiles converge with prior cluster-analytic and qualitative classifications of pregnancy weight attitudes into positive/accepting versus negative/concerned patterns (37, 51).

Dialogue with longitudinal research evidence. RI-CLPM studies demonstrate bidirectional, between- and within-person associations between weight self-stigma and exercise (38, 39). In that light, our 52.4% cross-sectional indirect association is likely an overestimate of the within-person longitudinal effect and should be interpreted as an associational, not causal, estimate. The direction attitude → internalized weight bias → avoidance is theoretically plausible and consistent with prospective work (37), but the reverse pathway and bidirectional relations cannot be excluded without repeated-measures data.

Why is higher concern the majority state? The majority status of the higher-concern profile likely reflects (a) intensive routine weight monitoring and risk-oriented counseling in Chinese antenatal care, which may inadvertently intensify weight anxiety; (b) pervasive “perfect pregnant body” and “gain weight only for the baby” narratives on Chinese social media; (c) historically heightened familial attention on the pregnant body following the one-child policy; and (d) possible limited scale discrimination at the high end of the response format. We recommend developing Chinese pregnant-women norms for the PWGAS and re-examining cut-off thresholds.

Cultural specificity of gestational weight concern in China. Several culturally specific mechanisms may shape gestational weight concern in this sample: the legacy of the one-child policy, which concentrated familial attention and anxiety on the single pregnancy and its body; the “zuo yue zi” (postpartum confinement) tradition and its associated dietary practices; elder-family (mother-in-law/mother) weight commentary and monitoring; and the “gain weight only for the baby, not for yourself” aesthetic narrative on Chinese social media. These factors help explain the majority-concern pattern and enhance the study’s novelty.

Importantly, internalized weight bias does not develop in a vacuum or solely from individual attitudes and beliefs. It is shaped by the social, cultural, healthcare, and structural contexts in which pregnancy is experienced (3, 58). In healthcare settings, routine weighing, risk-oriented counseling focused on gestational weight gain, and weight-centric care practices can, however unintentionally, communicate that body size is a matter of personal responsibility and moral worth. In the broader sociocultural environment, media narratives that idealize a “perfect pregnant body” and rapid postpartum “bounce-back,” together with family members’ commentary on weight and eating, constitute a pervasive climate of body surveillance. Within this climate, even women who begin pregnancy at the lower end of the BMI distribution may internalize the message that weight gain is a failure of self-discipline rather than a normal physiological adaptation. Consistent with a size- and weight-inclusive perspective, we therefore interpret weight-related concern in this study not as a deficit located within individual women, but as a response to—and internalization of—a broader weight-stigmatizing environment. This reframing has implications for practice: efforts to reduce exercise avoidance may be more effective when they address the weight-stigmatizing contexts that produce weight-related distress, rather than focusing solely on individual attitudes and behaviors.

### 4.2 Person-centered analysis

Using latent profile analysis, this study found that attitudes toward gestational weight gain among pregnant women could be divided into two profiles: the low concern profile and the high concern profile. This indicates that pregnant women’s attitudes toward weight gain are not simply distributed uniformly along a single continuous dimension; rather, there is relatively clear group heterogeneity.

The high concern profile accounted for a larger proportion of the sample, whereas the low concern profile accounted for a smaller proportion. In this study sample, a considerable proportion of pregnant women may have experienced relatively high levels of concern, worry, or emotional distress regarding gestational weight gain. Although gestational weight gain is a normal physiological process (54), weight change is visible and continuous, making it likely to be repeatedly noticed by pregnant women themselves, family members, and healthcare providers during weight gain.

When pregnant women lack sufficient knowledge about appropriate gestational weight gain, or are excessively influenced by appearance standards, postpartum recovery anxiety, and body-shape narratives on social media, they may be more likely to fall into the high concern profile. Therefore, in perinatal healthcare practice, healthcare professionals should not assume that all pregnant women can naturally accept weight gain. Nor should attitudes toward gestational weight gain be regarded as a single, stable, and homogeneous psychological state. Instead, the psychological needs of different types of pregnant women should be actively identified.

Further analysis showed that pregnant women in the high concern profile had significantly higher levels of internalized weight bias than those in the low concern profile. This suggests that types of attitudes toward gestational weight gain can effectively distinguish levels of internalized weight bias in pregnancy. Pregnant women in the high concern profile may be more likely to interpret gestational weight gain as appearance-related loss of control, failure of self-management, or nonconformity with social expectations, thereby experiencing higher levels of shame and self-denial.

In contrast, pregnant women in the low concern profile may be more likely to understand weight gain as a necessary physiological change during pregnancy and may show greater acceptance of bodily changes, resulting in less internalization of weight bias. This finding further supports the close relationship between attitudes toward gestational weight gain and internalized weight bias, and also suggests that programs addressing internalized weight bias should focus particularly on pregnant women who show high levels of concern or negative evaluation regarding gestational weight gain.

This study also found that pregnant women in the high concern profile had significantly higher exercise avoidance tendency than those in the low concern profile. This indicates that latent profiles of attitudes toward gestational weight gain can also identify risk in exercise behavior. Pregnant women in the high concern profile may be more likely to experience a sense of bodily exposure and being evaluated in exercise contexts. They may worry that others will notice their body shape changes, uncoordinated movements, or decreased exercise ability.

At the same time, they may associate exercise with weight control, body-shape management, or pressure for postpartum recovery, rather than understanding it as a positive behavior that improves pregnancy health and enhances physical comfort. This negative interpretation of the meaning of exercise may turn exercise situations into sources of stress, thereby increasing avoidance tendency. In contrast, pregnant women in the low concern profile may be less affected by internalized weight bias and body anxiety, and may be more likely to understand prenatal exercise as a means of health promotion and mind–body regulation.

### 4.3 Practical and clinical implications

The findings of this study have important practical implications for perinatal health care, prenatal physical activity promotion, and individualized nursing care. Health care providers should move beyond an exclusive focus on monitoring gestational weight gain and also consider pregnant women’s experiences, perceptions, and concerns related to body changes during pregnancy.

Rather than treating deviation from gestational weight gain recommendations as an independent trigger for referral, a person-centred model of care may be warranted, in which obstetric providers address weight-related concerns and provide guidance regarding physical activity and wellbeing during pregnancy. Any additional referrals should be based on identified clinical, nutritional, or psychosocial needs rather than gestational weight gain alone. Women experiencing significant body image concerns, weight-related distress, or internalized weight bias may benefit from psychological support, while nutrition support may be appropriate when concerns regarding nutritional adequacy or dietary intake are identified.

Because the present findings are cross-sectional, these suggestions are offered as directions for future investigation and clinical reflection rather than as evidence-based practice recommendations. Whether brief, low-burden assessment of weight-related attitudes and concerns could be integrated into routine antenatal care is an important question for future research. Within person-centred conversations, non-stigmatizing communication can help reframe weight gain as one aspect of overall wellbeing; for example, a statement such as “Your weight gain is too fast” might be rephrased as “Let us look together at how to keep a healthy rhythm for both you and your baby.” Support could also be tailored across pregnancy—for instance, normalizing bodily changes and addressing body image concerns in early pregnancy, supporting graded physical activity and countering social comparison in mid-pregnancy, and focusing on functional goals (sleep, back pain, labor preparation) and postpartum recovery expectations in late pregnancy. These possibilities require prospective evaluation before they can be recommended for routine practice.

### 4.4 Limitations and future directions

Despite the above findings, this study has several limitations. First, this study used a cross-sectional design. Although the mediation model was based on theoretical assumptions and statistical path testing, causal relationships among variables cannot be determined. Attitudes toward gestational weight gain may be associated with exercise avoidance tendency through internalized weight bias, but reverse pathways may also exist. For example, pregnant women with higher exercise avoidance may develop stronger weight anxiety and weight-related self-criticism due to reduced activity, faster weight gain, or increased physical discomfort. Therefore, future studies should adopt longitudinal designs and measure attitudes toward gestational weight gain, internalized weight bias, and exercise behavior at multiple time points, such as early, middle, and late pregnancy and the postpartum period, in order to examine the temporal sequence and dynamic changes among these variables.

Second, this study mainly relied on self-report questionnaires, which may be affected by social desirability, recall bias, and common method bias. Although Harman’s single-factor test suggested that serious common method bias was not present, this method can only provide preliminary evidence and cannot completely rule out common-source measurement error. Future research may combine objective physical activity measurement tools, such as accelerometers, wearable activity trackers, or mobile phone activity records, to assess pregnant women’s actual physical activity levels. Clinical weight records, antenatal care data, and healthcare provider assessments may also be incorporated to reduce bias caused by reliance on a single data source.

Third, the sample of this study was recruited from two hospitals in a specific region using convenience sampling, and the representativeness of the sample remains limited. The proportion of participants with a bachelor’s degree or above was relatively high, and the proportion of those reporting pregnancy complications was also relatively large, which may affect the generalizability of the findings. Future studies should expand sample sources by including pregnant women from different regions, different levels of medical institutions, urban and rural communities, and diverse socioeconomic backgrounds to improve the universality of the conclusions. In addition, comparisons across different cultural contexts may be conducted to examine differences in attitudes toward gestational weight gain and internalized weight bias in pregnancy, as well as the influence of sociocultural norms on body experiences during pregnancy.

In addition, the pre-pregnancy BMI distribution was heavily concentrated at the lower end of the BMI spectrum, resulting in underrepresentation of participants with higher BMI values. Consequently, the observed associations between pre-pregnancy BMI and weight-related concern should be interpreted with caution and may not generalize to populations with a broader BMI distribution. Future studies should recruit participants across the full BMI range to better understand the relationship between BMI and weight-related concern.

Finally, although the latent profile analysis identified two types of attitudes toward gestational weight gain, the naming and interpretation of the classes remain somewhat subjective. Different samples, profile indicators, or model specifications may produce different numbers of classes and class characteristics. The two-class model in this study was ultimately selected mainly on the basis of statistical indices, class proportions, and theoretical interpretability; however, more complex attitude profiles may still exist. Future research may replicate and validate the class structure identified in this study using larger samples. Further variables, such as body image, pregnancy anxiety, exercise self-efficacy, social support, experiences of medical weight communication, and social media use, may also be included to distinguish different psychological risk profiles in greater detail.

## 5 Conclusion

Based on cross-sectional survey data from 433 pregnant women, this study examined the relationships among attitudes toward gestational weight gain, internalized weight bias, and exercise avoidance tendency from variable-centered and person-centered perspectives. Pregnant women who held higher levels of negative attitudes toward gestational weight gain also reported higher exercise avoidance tendency, and this association was statistically mediated by internalized weight bias in cross-sectional analyses. Longitudinal research is needed to establish the temporal sequence and potential bidirectional nature of these relationships. The finding that the majority of pregnant women (73.21%) reported elevated weight-related concern suggests that non-stigmatizing, person-centred approaches to perinatal care—including attention to attitudes toward gestational weight gain—warrant further investigation; whether such approaches should be delivered universally or in a targeted manner is a question for future research and cannot be resolved by a cross-sectional design. Key future directions include longitudinal designs, objective physical-activity measurement (accelerometry), and multi-centre, representative sampling.

## Data Availability

The raw data supporting the conclusions of this article will be made available by the authors, without undue reservation.
