# Supplementary material for: Attitudes toward gestational weight gain and exercise avoidance tendencies: a latent profile analysis and mediation test of internalized weight bias in pregnant women
Source: Front Public Health. 2026 Oct 26;14:1944847. doi: 10.3389/fpubh.2026.1944847 (PMC13745249; doi:10.3389/fpubh.2026.1944847)
Supplement: Supplementary file 1 [file Table_1.docx]

# Attitudes Toward Gestational Weight Gain and Exercise Avoidance Tendencies: A Latent Profile Analysis and Mediation Test of Weight Stigma in Pregnant Women

Supplementary Table S1. Two-dimensional vs. unidimensional CFA of the PWGAS

| Model | χ² | df | χ²/df | CFI | TLI | RMSEA | SRMR |
| --- | --- | --- | --- | --- | --- | --- | --- |
| Unidimensional (16 items) | 983.28 | 104 | 9.45 | 0.35 | 0.33 | 0.140 | 0.125 |
| Two-dimensional (13 + 3) | 673.09 | 103 | 6.53 | 0.48 | 0.45 | 0.113 | 0.110 |

Note. Δχ² = 310.19, Δdf = 1, p < 0.001; interfactor correlation r = 0.516. These ML estimates (computed for the revision) reproduce the AMOS fit pattern; the AMOS 31.0 values for the unidimensional model (χ²/df = 2.953, CFI = 0.951, RMSEA = 0.067, TLI = 0.937) are retained in Table 1.

Supplementary Table S2. Mediation sensitivity: 16-item vs. 13-item attitude score

| Score | Total | Direct | Indirect | 95% CI (indirect) | Proportion |
| --- | --- | --- | --- | --- | --- |
| 16-item (primary) | 0.533 | 0.255 | 0.279 | [0.207, 0.358] | 52.4% |
| 13-item attitude only | 0.472 | 0.224 | 0.247 | [0.180, 0.323] | 52.4% |

Supplementary Table S3. Item means with SE and 95% CI for the two-profile solution.

| Items | Profile 1 (lower-concern) M (SE) | 95% CI | Profile 2 (higher-concern) M (SE) | 95% CI |
| --- | --- | --- | --- | --- |
| Proud of pregnancy look | 2.353(0.093) | 2.171–2.535 | 4.182(0.041) | 4.102–4.262 |
| Pregnant women beautiful | 2.319(0.095) | 2.133–2.505 | 4.141(0.038) | 4.067–4.215 |
| Like maternity clothes | 2.310(0.087) | 2.139–2.481 | 4.062(0.042) | 3.980–4.144 |
| Worry about getting fat | 2.256(0.090) | 2.080–2.432 | 4.208(0.039) | 4.132–4.284 |
| Careful about fat gain | 2.283(0.088) | 2.111–2.455 | 4.053(0.042) | 3.971–4.135 |
| Weight makes unattractive | 2.248(0.088) | 2.076–2.420 | 4.183(0.039) | 4.107–4.259 |
| Embarrassed when weighed | 2.197(0.085) | 2.030–2.364 | 4.182(0.036) | 4.111–4.253 |
| Bothered by stylish clothes | 2.093(0.085) | 1.926–2.260 | 4.167(0.044) | 4.081–4.253 |
| Embarrassed by size | 2.050(0.084) | 1.885–2.215 | 4.164(0.039) | 4.088–4.240 |
| Gain 35 lbs for baby | 2.491(0.107) | 2.281–2.701 | 4.157(0.036) | 4.086–4.228 |
| Gain 40 lbs for baby | 2.456(0.109) | 2.242–2.670 | 4.230(0.037) | 4.157–4.303 |
| Diet enough, don't care | 2.965(0.126) | 2.718–3.212 | 3.782(0.058) | 3.668–3.896 |
| Like gaining weight | 2.984(0.132) | 2.725–3.243 | 3.908(0.060) | 3.790–4.026 |
| Restrict after excess gain | 3.052(0.120) | 2.817–3.287 | 3.895(0.057) | 3.783–4.007 |
| Restrict to hide early bump | 2.896(0.121) | 2.659–3.133 | 3.848(0.059) | 3.732–3.964 |
| Skip eating before doctor | 2.544(0.124) | 2.301–2.787 | 3.844(0.055) | 3.736–3.952 |

Supplementary Table S4. Three-profile sensitivity solution

| Profile | Proportion | Interpretation |
| --- | --- | --- |
| Profile 1 | 26.32% | Lower concern (low on worry items, moderate acceptance) |
| Profile 2 | 63.99% | Higher concern (high on worry items) |
| Profile 3 | 9.70% | High concern / low acceptance (non-parallel shape) |

Note. LMR p = 0.981 (three-profile does not significantly improve on two-profile).
